# Collective constructions of ‘waste’: epistemic practices for disinvestment in the context of Dutch social health insurance

**DOI:** 10.1186/s12913-019-4434-1

**Published:** 2019-09-05

**Authors:** Floortje Moes, Eddy Houwaart, Diana Delnoij, Klasien Horstman

**Affiliations:** 1 Research School CAPHRI, Department of Health, Ethics and Society, Maastricht University, PO Box 616, 6200 MD Maastricht, the Netherlands; 2 Erasmus School of Health Policy and Management, PO Box 1738, 3000 DR Rotterdam, the Netherlands; 3 National Health Care Institute, P.O. Box 320, 1110 AH Diemen, the Netherlands

**Keywords:** Disinvestment, Administrative data, Low-value care, Epistemic practices, Ethnography, Dutch National Health Care Institute, The Netherlands

## Abstract

**Background:**

Faced with growing budget pressure, policymakers worldwide recognize the necessity of strategic disinvestment from ineffective, inefficient or harmful medical practices. However, disinvestment programs face substantial social, political and cultural challenges: mistrust, struggles for clinical autonomy or stakeholders’ reluctance to engage in what can be perceived as ‘rationing’. Academic literature says little about effective strategies to address these challenges. This paper provides insights on this matter. We analyzed the epistemic work of a group of policymakers at the National Health Care Institute on what was initially a disinvestment initiative within the context of the Dutch basic benefits package: the ‘Appropriate Care’ program. The Institute developed a strategy using national administrative data to identify and tackle low-value care covered from public funds as well as potential underuse, and achieve savings through improved organization of efficiency and quality in health care delivery. How did the Institute deal with the socio-political sensitivities associated with disinvestment by means of their epistemic work?

**Method:**

We conducted ethnographic research into the National Health Care Institute’s epistemic practices. Research entailed document analysis, non-participant observation, in-depth conversations, and interviews with key-informants.

**Results:**

The Institute dealt with the socio-political sensitivities associated with disinvestment by democratizing the epistemic practices to identify low-value care, by warranting data analysis by clinical experts, by creating an epistemic safe space for health care professionals who were the object of research into low-value care, and by de-emphasizing the economization measure. Ultimately, this epistemic work facilitated a collaborative construction of problems relating to low-value care practices and their solutions.

**Conclusions:**

This case shows that – apart from the right data and adequate expertise – disinvestment requires clinical leadership and political will on the part of stakeholders. Our analysis of the Institute’s Appropriate Care program shows how the epistemic effort to identify low-value care became a co-construction between policymakers, care providers, patients and insurers of problems of ‘waste’ in Dutch social health insurance. This collective epistemic work gave cognitive, moral and political standing to the idea of ‘waste’ in public health expenditure.

**Electronic supplementary material:**

The online version of this article (10.1186/s12913-019-4434-1) contains supplementary material, which is available to authorized users.

## Background

The desire to maintain more sustainable health care systems is placing growing pressure on budget-holders, worldwide, to reassess currently funded medical practices and potentially release resources to higher priority areas [1, 2]. Policymakers recognize the necessity of complementing cautious investment in new technologies with strategies to reduce the use of unnecessary, ineffective, inefficient or harmful care that is currently used in practice [3–5]. The term ‘disinvestment’ refers to processes of ‘partially or completely withdrawing health resources from existing healthcare practices, procedures, technologies or pharmaceuticals that are deemed to deliver little or no health gain for their costs, and thus do not represent efficient health resource allocation’ [6]. Disinvestment takes different forms ranging from the full withdrawal or substitution of services to the restriction of services due to inappropriateness of use or savings achieved through better organization of efficiency in care practices [7, 8]. Reducing expenditure on ‘low-value care’ – i.e. ‘interventions where the risk of harm or costs exceeds the likely benefit for a patient’ [4] – allows investment in higher valued care and in this way increases the efficiency of a health care system. Though low-value care practices seem obvious candidates for disinvestment, the withdrawal of resources from existing medical services unavoidably raises ‘political and professional complexities’ as it is associated with ‘restrictions on clinical autonomy and patient choice’ [9].

It is commonly assumed that evidence of a medical treatment’s (in)effectiveness or data on inappropriate use can provide a factual, medical–technical base for thorny (dis)investment decisions (see e.g. [10, 11]). One way to assess low-value care is by looking at routinely collected administrative data [12–15]. Administrative datasets containing information about health insurance claims, hospital admission, drug prescriptions et cetera provide population-level insights on service utilization [12]. Low-value care can be found by comparing administrative data recording what *actually* happens in health care practice with, for example, the *norms* for practice as stated in (evidence-based) professional standards and guidelines. However, studies have shown that, despite the use of evidence to identify low-value care, disinvestment programs often face substantial social, political and cultural challenges [1, 5, 6, 11].

For example, disinvestment initiatives can encounter resistance to change due to clinicians’ established medical training and practice paradigms [6] and due to clinicians’ unwillingness to engage in what is easily perceived as ‘rationing’ [1]. Disinvestment can lead to stakeholders’ feelings of disempowerment and the idea that restrictive policies are ‘imposed’ on them by external agents, especially when stakeholders have no opportunities to ‘influence draft policies’ or are presented with ‘near finalized’ plans [1]. The idea that external non-medical agencies have something to say about medical practice challenges clinicians’ particular ownership of medical knowledge and is often associated with restrictions to clinical autonomy [1, 6, 9]. Effective disinvestment thus relies not only on sound technical reasoning, but also on an understanding of social values [11], on local and national relationships [16] and on ‘political will’ [6]. Literature on disinvestment therefore highlights the importance of inclusive dialogue, stakeholder involvement and transparency to create collaborative support for disinvestment initiatives [1, 5, 9, 11, 16–20].

While policymakers are generally aware of the fundamental importance that ‘disinvestment is not perceived as a blunt, all-or-nothing instrument of rationing’ [6, 9], both clinicians and policymakers often experience that they are ‘operating within an environment of mistrust’ [1]. While policymakers fear that clinicians might be biased by potentially reduced revenues, clinicians, in their turn, fear that they might be wrongfully blamed that their practice is ‘skewed by financial gain’ [1]. Mistrust and blame can hinder collaboration; inclusive dialogue and trust are seen as key aspects of successful disinvestment [1, 5, 9, 11, 16–20]. In academic literature little has been written about effective practices of realizing ‘trust’ and (ultimately) collaborative support for disinvestment. This paper provides insights in this regard and examines how the epistemic practices of policymakers can help to establish trust and collaboration in disinvestment initiatives.

Drawing on ethnographic research in Dutch practice, we examined the epistemic practices of a group of Dutch policy advisors working on what was initially a disinvestment initiative within the context of the Dutch basic health insurance benefits package. The National Health Care Institute (*Zorginstituut Nederland)* is an independent governing agency that falls under the responsibility of the Ministry of Health. The Institute is tasked with the management of the basic benefits package in the Netherlands, and is lawfully responsible for the organization of public information about the quality of care. In 2014, this Institute introduced a program called *Zinnige Zorg* [hereafter translated as ‘Appropriate Care’] that aimed to improve the quality of care funded from Dutch basic health insurance and save unnecessary public expenditure by identifying (and ultimately removing) ineffective and inappropriate procedures. The Institute’s authoritative position in the Dutch health care system and its exclusive access to three national administrative datasets allowed the Institute to recognize low-value care practices and identify potential candidates for disinvestment. How did the Institute – in its epistemic practices – deal with the socio-political sensitivities associated with disinvestment?

We first introduce the Dutch health care system, the role of the National Health Care Institute within this system and the Appropriate Care program. We then elaborate on our theoretical background on epistemic practices and our research methods. In the results section, we analyze the Institute’s epistemic practices in the Appropriate Care program and how the Institute dealt with trust and the socio-political sensitivities associated with disinvestment by means of their epistemic work.

### Dutch health insurance system and the National Health Care Institute

In the Netherlands, standard basic health insurance is provided for all citizens by private health insurance companies. The 2006 Health Insurance Act (*Zorgverzekeringswet*, Zvw) obliges everyone who resides – or pays payroll tax – in the Netherlands to take out basic health care insurance from a private insurance company. Citizens can choose between competing health insurance companies during a yearly open enrollment period. Income-related subsidies make basic healthcare insurance affordable for all citizens. Insurers are obliged to accept enrollees regardless of their age or health condition, while a risk adjustment scheme compensates them for clients with predictably high medical expenses [21]. Private insurance companies are expected to negotiate the prices, services, and quality of care with health care providers on behalf of their insured clients [22].

The Minister of Health formally requests advice from the National Health Care Institute to specify treatments, drugs and medical aids that are reimbursed from the basic health insurance and those that are not. The Institute subjects major new drugs to health technology assessments (HTA), thereby systematically evaluating the drugs’ (cost-) effectiveness, safety, ethical, social and financial issues, before coverage is considered. With regard to existing medical treatments and non-pharmaceutical interventions, the Institute issues authoritative standpoints whenever there is a lack of clarity amongst stakeholders about the coverage of a treatment, drug or medical aid.

### The appropriate care program

The Dutch government’s Coalition Agreement in 2012 included a total cutback of approximately €300 million euros on basic health insurance, which was to be largely realized by a systematic screening of the basic benefits package. In 2013 the Minister of Health announced that this screening was to be carried out by the National Health Care Institute and that its goal was to stimulate appropriate use of the basic benefits package and identify potential savings [23]. In response, the National Health Care Institute set out to develop a new approach to package management: the Appropriate Care program.

‘The main aspects of package management as currently implemented [HTA and reimbursement standpoints] are still important, but are merely preventing redundant, extra growth in costs […]. The Appropriate Care (*Zinnige Zorg*) program was started in 2014 […] to propose a new way of increasing the quality of care while economizing on costs; the key to this is identifying and reducing ineffective and/or unnecessary care’ [24].

A team at the National Health Care Institute started developing a program to screen the basic benefits package for low-value care by systematically combing through the package of provisions in a cyclical five-year process, addressing each of the 10 domains of the International Classification of Diseases (ICD-10).

Due to its unique position within the Dutch health care system [25], the Institute has exclusive access to three national administrative datasets: GIP data, DIS data and Vektis data. The GIP-database [Medicines and Medical Aids Information Project, GIP] contains prescription-related data on the use of drugs and medical aids. The DIS-database [Claims Information System, DIS], maintained by the Dutch Healthcare Authority (*Nederlandse Zorgautoriteit*), contains detailed information about all care trajectories carried out in hospitals, mental health organizations and rehabilitation centers. The Vektis-database (managed by the data specialist of Dutch health care insurers) covers a wider range of data including care for the elderly and paramedical care.

**Fig. 1:**
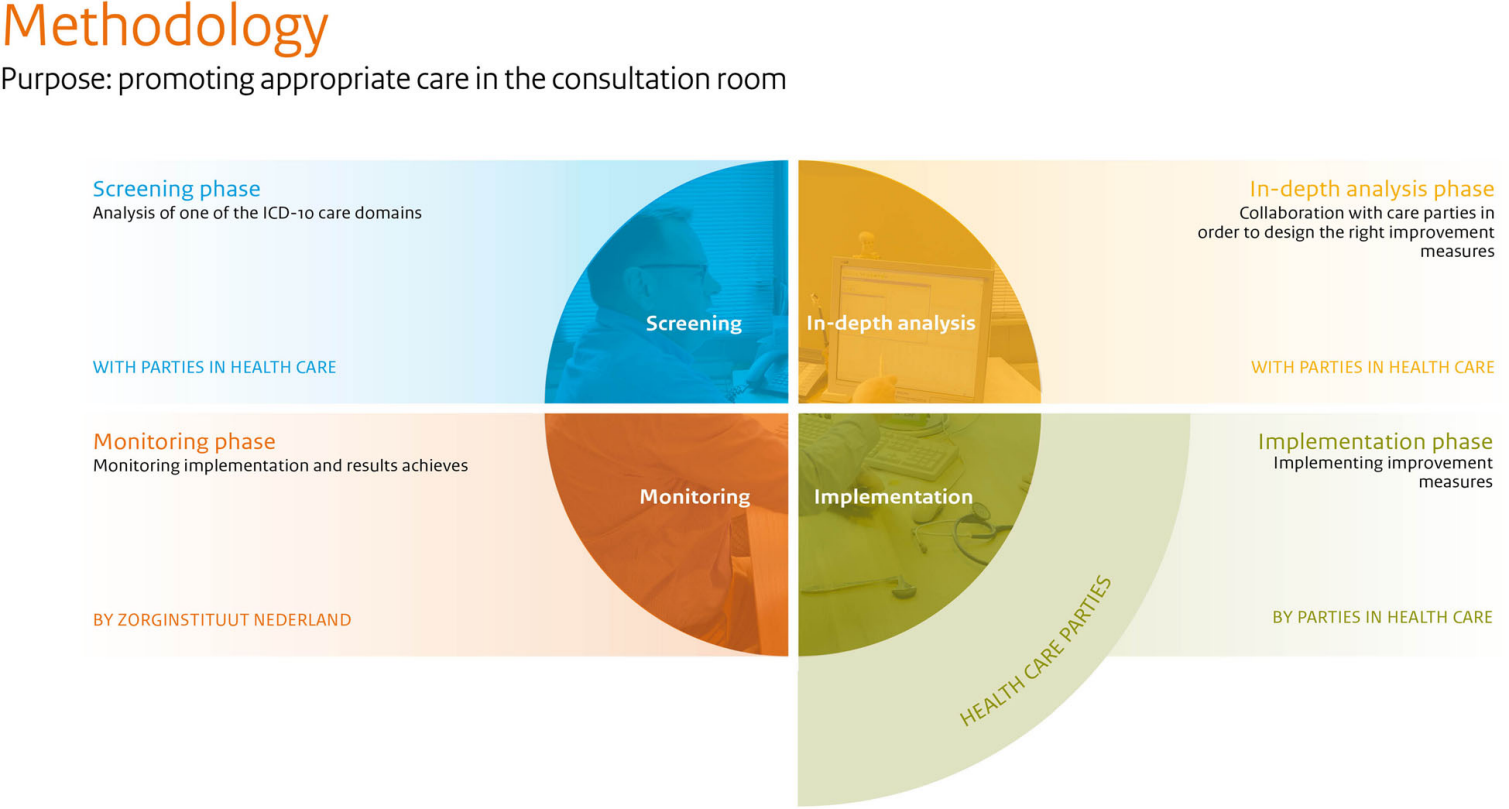
Methodology of the Appropriate Care Program

The Appropriate Care program consists of four phases in which low-value care is analyzed and tackled [see Fig. 1]. First, in a ‘Screening Phase’, the Institute screens an ICD-10 domain for potentially low-value care. For indications of low-value care, the Institute consults different professional groups and sorts through professional media, patient-reported outcome measures (PROM), consultancy reports and scientific research on low-value care. The Institute also provisionally checks administrative data for matters that can indicate the inappropriate use of care, such as practice variations, massive increases or decreases in costs, extended length of hospital stay or high rates of adverse events, readmissions or re-operations. The screening phase results in a ‘screening report’, containing a set of topics for further research per ICD-10 domain. To date, six screenings have been finished: Diseases of the Circulatory System (ICD-10 IX100–199); Neoplasms (ICD-10 C00-D84); Diseases of the Respiratory System (ICD-10 X (J00-J99); Diseases of the Nervous System (ICD-10 VI (G00-G99)); Mental and Behavioral Disorders (ICD-10 V (F00-F99)); Diseases of the Genito-urinary System and Pregnancy, Childbirth and the Puerperium (ICD-10: XIV (N70-N98) and ICD-10: XV (O00-O99)).

Second, in an ‘In-depth Analysis Phase’, the Institute conducts in-depth analysis into the selected topics of low-value care. The Institute defines what is considered by the medical community to be ‘appropriate’ care for a diagnosis as specified, for example, in clinical guidelines and quality standards. It subsequently compares these norms to the actual care delivered in practice, by conducting a targeted analysis of the administrative data of the types of care registered and paid for in practice. This phase results in a ‘room for improvement’ report. In the ‘room for improvement’ report, the Institute proposes actions to improve care and it includes an estimation of the costs that can be saved in these practices. Furthermore, the Institute formulates agreements with health care professionals on how to improve the quality of those medical practices and ultimately save costs. So far, seven ‘In-depth’ analyses have been completed: care for Peripheral Artery Disease (PAD), care for chest pain (suspected stable angina pectoris), post-treatment surveillance of women treated for breast cancer, care for Castration-Refractory Prostate Carcinoma (CRPC), expensive oncolytics (appropriate care using expensive pharmaceuticals for metastatic renal cell carcinoma), end of life care for people with incurable intestinal cancer or lung cancer, care for arthrosis of the knee or hip.

Third, in the ‘Implementation Phase’, field parties – in dialogue with the National Health Care Institute – implement the health care improvement measures that were planned in the ‘In-depth Analysis Phase’. Fourth, in the ‘Monitoring Phase’, the Institute monitors the progress made in the implementation of improvement measures and the results of these efforts. Seven topics are currently being implemented and monitored: PAD, chest pain, post-treatment surveillance of women treated for breast cancer, CRPR, expensive oncolytics, end of life care for incurable intestinal cancer or lung cancer, arthrosis in knee or hip. None of these topics have yet completed the Implementation and Monitoring Phases.

In order to identify low-value care, the Appropriate Care program works from the patients’ perspective and uses ‘Lean Thinking’: following the patient as he/she goes through the health care system, aiming to identify value-added and non-value-added steps in the care process (see [26]). The focus in the Appropriate Care program is not on scraping services or technologies in their totality, but rather on targeting their (low-value) use for certain patients or in certain medical situations. Typically, the outcome of an Appropriate Care trajectory involves recommendations and tools for better guideline compliance, stepped care, shared decision-making, development of quality information (outcome indicators) and improved patient information (a similar approach to the ‘Choosing Wisely’ Campaign [27]). [See Table 1 for two examples of Appropriate Care trajectories].

**Table 1:** Two examples of Appropriate Care trajectories

Phase	Topic	
	Knee/Hip arthrosis	Peripheral Artery Disease (PAD)
Screening	The first topic selected for an Appropriate Care trajectory did not result from a screening, but was initiated by the Ministry of Health. Based on reports showing medical practice variations in diagnostic interventions and prosthesis placements in care for hip and knee arthrosis and PROMs reporting that 1 in 5 patients was not satisfied with the outcome of the treatment received for hip and knee arthrosis, the Ministry of Health suggested ‘care for patients with hip or knee arthrosis’ as the first topic for the Institute to research within the context of the Appropriate Car program.	In 2015 the Institute published its Screening report of Diseases of the Circulatory System (ICD-10 IX100–199). The Institute selected 3 topics for further research: Implantable Cardioverter Defibrillators, Stable Angina Pectoris and Peripheral Artery Disease (PAD). PAD was selected for 2 reasons: 1) PAD stage 24 (also known as Claudicatio Intermittens or ‘window-shoppers’ disease’) was marked by stakeholders as a topic that required the attention of the National Health Care Institute; 2) screening identified PAD as a topic for further research based on data on costs, volume and their growth.
In-depth analysis	In-depth analysis showed that [28]: 1. Too many patients receive an X-ray, MRI, arthroscopy or puncture for diagnostics, while clinical guidelines state that these are only needed in exceptional cases. The Institute estimates that 90% of the diagnostics are not necessary. 2. In current clinical guidelines and in daily clinical practice little attention is paid to shared decision-making and stepped care, while this is thought to lead to a decrease in the number of surgical interventions and a more selective placement of prostheses. The Institute estimates that this could potentially lead to a reduction of prosthesis placement of 10% for knee prosthesis and 5% for hip prosthesis. 3. PROMs for placement of knee and hip prosthesis are available, but require further development and validation to provide insights into actual health outcomes and require further definitions of patient characteristics that are related with an unfavorable outcome of the prosthesis. Actions for improvement: • guideline compliance for the use of (imaging) diagnostics • More selective placement of knee and hip prostheses • Further development of PROMs Estimated budget impact: 49 million euros	In-depth analysis showed that [29]: 1. Too many patients are referred to a vascular surgeon for diagnostics, while clinical guidelines indicate that ankle-brachial pressure index diagnostics can be performed under the responsibility of a GP. 2. 11,000 unnecessary duplex ultrasounds a year are carried out, while clinical guidelines state that duplex ultrasound should only be used if endovascular revascularisation (ER) or surgery are being considered 3. 75% of patients received no Supervised Exercise Therapy (SET) as first-line treatment and 20% of patients may undergo ER unnecessarily, while guidelines state that all new patients with PAD should get SET as first-line treatment. Actions for improvement: • Improved agreements between care professionals • Provision of reliable patient information • Development of quality information • Inform GPs that diagnostics can be outsourced to a vascular laboratory without referral to a specialist • All newly diagnosed patients receive SET as first-line treatment. • Reimbursement of SET • Aim to go from 35 to 11% of patients undergoing ER Estimated budget impact: 30 million euros
Implementation	The following concrete actions are implemented by field parties to improve the care for patients with knee/hip arthrosis: • Offering of patient information on knee/hip arthrosis (and its treatment) on one central website (Thuisarts.nl) • public availability of PROM • revision of the GPs guideline on ‘non-traumatic knee problems’ (by the Dutch College of General Practitioners (NHG)) • development of the multidisciplinary guideline ‘conservative treatment of knee/hip arthrosis’ including transmural stepped care agreements (by Dutch Orthopedic Association (NOV)) • development of the guideline ‘Total Hip prosthesis’ (by NOV) • Development of the guideline ‘Arthrosis Hip/Knee’ (by the Royal Dutch Society for Physical Therapy (KNGF))	These are a few examples of the concrete actions that are being implemented by field parties to improve care for patients with PAD: • The organization of ankle-brachial pressure index diagnostics in primary care will be improved. Information will be made available for primary care professionals on the possibility of having these diagnostics carried out in primary care diagnostic centers and vascular laboratories. Accessibility of the latter will also be improved (by the NHG, the Netherlands Association of Surgeons (NVvH), together with the National Health Care Institute) • Clear agreements will be made between primary care and hospital care about advice on diagnostics and treatment (NHG), the Netherlands Association for Vascular Surgery (NVVV), together with other relevant parties) • Patient information must improve, e.g. by offering reliable patient information in a single location. (The Heart&Vascular Group, NHG, NVvH) • Attention should be given to stepped-care, i.e., explaining properly why an operation is not the first choice (by all professionals). • Et cetera
Monitoring	Evaluation and monitoring of the implementation of improvements is planned for 2019.	Evaluation and monitoring of the implementation of improvements is planned for 2020.

### Research aim

The widely reported social, political and cultural challenges to disinvestment [1, 5, 6, 11] hold particularly true for the Dutch context. Clinicians traditionally enjoy strong professional autonomy in the Netherlands and government interference in health care remains a sensitive issue [30]. Due to the corporatist structure under which the system of social health insurance operates, the Dutch have a consensus-building form of policymaking called ‘poldering’ that requires stakeholder engagement [25, 31]. Though the National Health Care Institute is an authority in the field of social health insurance, at the same time, it occasionally encounters distrust from field parties. As manager of the basic benefits package, the Institute is easily perceived as an ‘instrument of the Ministry of Health to cut health care expenditure’ [30]. This paper explores how the Institute dealt with the delicate issue of balancing its own authoritative position in the field with clinicians’ professional autonomy and the issues of (dis)trust that are typically associated with disinvestment efforts. This paper centers around the question of knowledge: how did the Institute deal with the socio-political sensitivities associated with disinvestment *by means of their epistemic work*?

## Methods

### Theoretical background

Science and Technology Studies (STS) [32] is a field of research that takes an empirical approach to studying knowledge in the daily practices of scientists, engineers and other professionals [33, 34]. Every place that produces knowledge claims is, according to Knorr Cetina, host to ‘practices, arrangements and mechanisms’ which – in that field of professional expertise – make up ‘how we know what we know’ [35]. Such working methods, techniques or tools of knowing express an epistemic ideal of ‘objectivity’ in professional life [36]. Ultimately, epistemic practices are related to how a profession makes itself publicly accountability for what it is licensed to do [36]. Policymakers, too, have their own ‘epistemic culture’ [37], and public accountability for policy choices is constituted in these particular epistemic practices [36].

This paper draws from 4 years of (etic) ethnographic research conducted at the National Health Care Institute. We studied the epistemic practices of ‘how knowing is done’ [38] at the National Health Care Institute. In particular, we looked at the Institute’s practice of creating and warranting knowledge as a basis to justify policy decisions. Due to the public nature of the Institute, its knowledge work centers on the justification of decisions. What types of knowledge (e.g. experimental evidence, expertise, experience of doctors and patients) are used by the National Health Care Institute in its efforts to attain impartiality, objectivity and justice in policy decisions?

In this paper we report on the epistemic practices of the Institute in the Appropriate Care program to understand how knowing was done and how legitimation and justification was achieved in the context of this program. As is customary in the field of STS, we refrain from taking a stance on what kind of knowledge should be used to attain impartiality, objectivity or justice in decision-making. Abstaining from taking a standpoint, allowed us to conduct systematic research into the way others ascribe meaning, truthfulness, political significance and moral weight to particular forms of knowing.

### Data collection

It is in the Institute’s daily practices that public accountability is ‘done’ by justifying and legitimizing public policy choices by drawing on specific working methods. We had the opportunity to study these daily practices by conducting intensive ethnographic fieldwork and documentary research at the National Health Care Institute, between October 2013 and September 2017. This research was done in the context of a project called ‘Public Legitimation of Social Healthcare Insurance in the Modern Risk Society’, funded and facilitated by the National Health Care Institute and independently executed by researchers of Maastricht University (see funding section at the end of this paper for specificities).

In this research project we selected several concrete examples of the Institute’s knowledge-intensive decision-making practices and studied how public legitimation was constituted in these epistemic practices (see [39, 40]). As the Institute’s Appropriate Care program was a ‘flagship’ project for the Institute, we decided to focus part of the study on this program. This paper reports on that particular case-study. In collecting and analyzing data for this study, we used the ethnographic methodology previously described in the context of this research project [39, 40].

Empirical data were collected by the first author through in-depth conversations, document analysis, selective overt non-participant observation of meetings, semi-structured open interviews and focus groups. Findings were recorded in daily field notes. Following the same protocol used for other case-studies [39, 40], the first author familiarized with the Appropriate Care program through informal conversations with staff members and Institute directors and by studying written material on the program (including emails, minutes of meetings, official and informal documents). She, then, gained direct experience with the program’s epistemic practices by attending (three) team meetings, (seven) executive board meetings and (six) meetings of advisory committees in which the program was discussed. Ultimately, findings were triangulated in (three) interviews and (two) focus groups. Interviewees were selected through ‘purposive’ sampling of information-rich informants [41]. We selected interviewees who held information about the political, social and technical aspects of the epistemic work conducted in the context of the Appropriate Care program. We carried out semi-structured open interviews based on a topic list with three key informants affiliated with the Appropriate Care Program. We, furthermore, held two focus groups, for which we selected interviewees who were involved explicitly in the epistemic work regarding the Appropriate Care program (technical analysis, data analysis, et cetera). See Additional file 1 for a sample of the topic list (developed for this case study) that was used for the interviews and focus groups. All interviews were audio-recorded, after receiving the informants’ consent, and transcribed in full.

### Data analysis

The analysis presented in this paper focuses on the epistemic work relating to the ‘Screening Phase’ and the ‘In-depth Analysis Phase’ of the Institute’s Appropriate Care Program. We focus on these two phases because the working methods for these two phases were developed during our field work. Methods for the ‘Implementation Phase’ and the ‘Evaluation Phase’ were still in development when we were rounding off our fieldwork so they could not be included in our analysis.

The data were analyzed as follows: the first (FM), second (EH) and fourth (KH) authors engaged in an iterative process of joint close reading of field notes, reports, policy documents, minutes and interview transcripts (that were manually coded and analyzed). The third author (DD) engaged in discussions of draft versions of this paper.

The leading questions in the analysis were: What data, information and knowledge sources were used by the Institute in their effort to identify low-value care? How was the process of data analysis organized? Who was involved in formulating research questions and interpreting the results of these inquiries? How were data analyses - that identified low-value care practices - made public and actionable? In finding answers to these questions, we aimed to understand how the Institute dealt with socio-political sensitivities associated with disinvestment by means of their epistemic work. As a ‘member-check’, we sent a written version of the analysis presented in this paper to staff members involved and interviewees to test our analysis on them.

## Results

### Warranting data analysis by clinical experts

The first focus group revealed that the basic idea of the Appropriate Care program was to identify low-value care by comparing what was regarded by the medical community as good practice (as specified for example in clinical guidelines and quality parameters) with care actually delivered in practice (for example by looking at administrative data on the treatments registered and paid for in practice). One of the participants in the focus group explained:

“I think that the real point of departure has always been, what do those who work in the field regard as good care? And where have they recorded this, e.g. in guidelines, or possibly in the quality parameters that they generally use? (…) and the second question is: is this how it is done in practice? (…) For example, we all feel that, before operating on *claudicatio intermittens*, you first determine whether physiotherapy helps, don’t you? This is called ‘running training’. Then you look at the [reimbursement] data: how many people have had the running training?” (focus group#1).

If patients did not receive physiotherapy or ‘running training’ before undergoing an operation, this could be an indication of low-value care, because patients are unnecessarily subjected to invasive surgery.

However, our field notes about Appropriate Care program meetings showed that the technical analysis of administrative data was no easy task. To illustrate this, we show an excerpt from our field notes below. These field notes were taken at a meeting between the Appropriate Care program team and two external data consultants. During the meeting, the group looked at administrative data on the treatment of lung cancer and investigated the hypotheses that care for lung cancer patients in the follow-up treatment after initial surgery was inefficient, and thus, low-value care. The group tried to make sense of the surprisingly low number of lung cancer patients who were actually in follow-up trajectories. The first author recorded some of the questions that were raised with regard to the administrative data:

“the survival is only 20% … maybe this explains the low numbers?…which patients should be selected? …would it be a mistake to focus on those who underwent an operation? … this dataset does not allow us to see who died... the number of people who do not return for a follow-up treatment – are they the ones who died?” (unpublished observation FM).

The Institute found that, in order to interpret the data accurately it was necessary to closely involve clinical experts who knew not only clinical guidelines, but also understood clinical practice as well as administrative practices. Therefore, the Institute hired clinical experts at the Institute to help conduct data analysis:

“We hire knowledge experts from the field who spend four days a week operating, and one day cooperating with us. In fact, we can learn an awful lot from them. [A clinical expert can do] a spot of brainstorming with us and he/she can demarcate our populations and say: ‘well, if you are looking at this treatment it would be nonsensical not to look at that treatment as well...’” (focus group#1)

From our focus groups it became clear that the in-house availability of clinical expertise facilitated the Institute to carry out a better technical data analysis of low-value care. Focus group participants suggested that there was also an additional socio-political advantage to the close involvement of clinical experts. Data analysis performed only by non-medical parties – “people who have studied business administration” or “blue suits” (focus group#1 and 2) – often lack the trust of field parties. Focus group participants suggested that the fact that data analysis was performed with the aid of clinical experts generated a form of trust that policymakers and data experts alone did not have.

### Epistemic participation of stakeholders

The first topic that was investigated in the context of the Appropriate Care program was whether patients with arthrosis in hip and knee were receiving appropriate care. From our documentary research and interviews it became clear that the Ministry of Health suggested this topic to the Institute, based on PROMs showing that one out of five patients claimed no improvement after a hip or knee prosthesis and on a report of medical practice variations in treatment of knee and hip arthrosis [42]. One of our interviewees recalled: “it seemed like a really good topic to tackle immediately” (informant#3).

However, according to interviews and focus groups, the Institute found that orthopedists were surprised that the Institute was investigating the appropriateness of care for knee and hip arthrosis as orthopedists had not played any significant part in selecting this topic for screening. Partly in response to this event, and partly because stakeholder involvement is typically part of the Institute’s style of decision-making, the Institute decided to consult field parties systematically in the process of selecting research topics in the Screening Phase. In subsequent Appropriate Care trajectories, field parties had the opportunity to bring topics to the table that seemed important based on their experience and perspectives.

Not only the screening for topics of low-value care was done in negotiation with field parties, stakeholders were also involved in the subsequent epistemic work of designing relevant research questions. An Appropriate Care trajectory now starts with a ‘kick-off’ meeting that gathers representatives of all relevant parties at one table. The focus group revealed that stakeholders are involved in the epistemic practices of the Appropriate Care program right from day one:

“formulating the research question, in the sense of involving the parties as well, ... i.e., which questions need to be answered by the data. In other words, involving them up front, and then setting to work and finally checking the results with them” (focus group#1).

From our interviews it became clear that the participation of stakeholders was both a way to include the knowledge of field parties in screening for low-value care, as well as a way to address issues of trust. An interviewee revealed that the participation of stakeholders was all about “having increasing confidence in one another” (informant#1). In our field notes of an executive meeting, we recorded someone saying on the matter of stakeholder participation that it is best “to involve them [field parties] in smaller steps and allow dominant choices to be partly theirs too” (unpublished observation FM).

This did not mean that the Institute left choices, for example of screening topics, entirely up to stakeholders. Focus group participants explained: “where are the most important topics? In answering this, of course, we look at what impact we can have” (focus group#2), for example practices with a high volume of patients or high costs, as this would potentially yield the highest gains in health quality and save public costs. Working from the perspective of a public governing agency, the Institute does prefer some topics over others: “we introduce societal interests, so this gives us a strong position at the table” (focus group#1).

### Creating an epistemic safe space

The use of national administrative data allowed the Institute to map care that was registered and paid for in practice. Our interviews revealed that these data allowed a wide range of analyses:

“You can in fact combine everything at hospital level…. or you can do it based on time: e.g. how many operations were carried out on that day? or you can do it at patient level: e.g. what happened to the patient over the course of time?… you can do an awful lot with this [these data]” (informant#2).

Reimbursement data, however, is privacy-sensitive information. From our interviews it became clear that the Institute was very cautious in handling and publishing these data. One of our interviewees commented that “care providers or hospitals allow you to see something of what is going on behind the scenes… which means they are to a degree relinquishing some of their power” (informant#2). One of the lessons that the Institute learned in the context of the Appropriate Care program was that “handling data correctly” (informant#2) was very important, especially when publishing data that could be traced back to specific institutions.

The interviewee explained that, while it is broadly accepted “that you do not publish data on patients”, still a topic of public debate is whether this is also the case for “publicly funded institutions” (informant#2). As Dutch citizens spend so much public money on health care, it might be appropriate that everyone in society is actually allowed to see the aggregate result of “all those invoices on which we are all spending our [public] money” (informant#2). The publication of e.g. data about practice variations can be relevant, furthermore, for both patients and insurers, as it conveys information about the quality of care provided in different hospitals. Despite the fact that it was interesting from a public (accountability) perspective to publish data on practice variations, the Institute never published hospital-specific or provider-specific information in any of its reports. Our interviews showed that the Institute actively avoided the publication of data that could be traced back to specific institutions in order to create a “safe environment” for parties:

“you have to conduct the substantive discussion in a safe environment […] If we were to immediately publish reports painting the full picture of everything that goes wrong, then the discussion would change entirely, as everyone would crawl back into their shell” (informant#1)

Our documentary research revealed that the professional media debated the Institute’s decision not to publish medical practice variation data on care for cancer at an institutional level, and blamed the Institute for lack of transparency and listening too closely to the interests of care providers [43]. The National Health Care Institute, however, claimed that they did not have a mandate to publish institution-specific data [44]. Our interviews showed that, while the publication of institute-specific data could be a strong tool for holding professionals accountable, for the Institute it was important that things did not get personal in order to keep field parties involved. One interviewee commented: “if it isn’t necessary… then we do not reveal information about care providers… [it is a matter of] balancing the power of communication versus […] unnecessary disclosure” (informant#2). In order to keep the stakeholders on board, the Institute conducted their epistemic work involving privacy-sensitive information about low-value care in an environment that was safe for those who were the object of that inquiry.

Our focus group reported that the Institute did organize multi-stakeholder meetings in which findings were discussed on a general level (i.e. not institute-specific). At these meetings professional groups started “asking one another questions” (focus group#1). For example, when research showed that an “awful lot of arthroscopies were carried out for arthritis”:

“then patient [organizations] started saying: ‘That’s not a good thing at all, not if it’s not doing anyone any good’… And, then the physiotherapists said: ‘Yes, those patients are far better off going to a physiotherapist’ […] If you speak to the parties individually, then they could say: ‘Yes, but GPs are too quick to refer, you shouldn’t be talking to me, but to the GPs’... However, when they too are at the table, then you see that things are expressed differently and possibly also that people start saying: well, yes, perhaps we also played a role here…” (focus group#1)

As such, professional groups were involved both in interpreting the study results and in formulating potential solutions for low-value care problems, without having to fear potential cutbacks or reputational damage for their own specific institutions.

### Shifting the aim of epistemic work

The Appropriate Care program was initiated after the Minister of Health announced that the National Health Care Institute would be carrying out a systematic screening of the benefits package in order to realize budget cuts in the context of social health insurance. This screening would identify low-value care practices currently covered by the basic benefits package. Our interviews and focus groups identified resistance to economizing or ministerial budget cuts on the part of those working at the Institute. The Institute did not want the Appropriate Care Program to be directly associated with disinvestment. In our field notes we recorded the following concern that was expressed in an executive meeting:

“enormous pitfall in the entire trajectory… the link with economizing; although this is what the Minister requested… we do not carry out economization tasks, we look at quality and effectiveness” (unpublished observation FM)

From our interviews it became clear that the principle goal of those working for the Appropriate Care program was ensuring that patients received the right care at the right time in the right way. The idea was that this would save money in the long run (even though it might entail investment in the short term). Focus group participants revealed that “naturally, we do have to choose topics that we feel will eventually result in real economies…” (focus group#1). Yet, in their epistemic work – in formulating hypotheses, research questions and interpreting data analysis – the Institute put the emphasis on quality and safety concerns before economization. The focus group participants explained that, in the Appropriate Care Program: “we [have] always thought that if you promote quality, the euros would follow (…) the focus is on improving quality (…) we weren’t trying to remove provisions from the benefits package” (focus group#1). The Institute treated economization not as the main goal of their epistemic work with regard to low-value care, but as a spin-off to organizing more efficient, high quality and safe health care.

From our observations, interviews and focus groups, it became clear that the reason to push the aim of economization to the background was also related to issues of trust. One interviewee revealed, for example, that talks with field parties always focused first on how to improve the efficiency, safety and quality of low-value care practices, and only thereafter “we chart what actual [financial] effect these [improvements] will have; and this … is sensitive, because the parties involved fear that they will be short-changed” (informant#3). In order to keep stakeholders on board in the Institute’s effort to identify and tackle low-value care practices currently covered from basic health insurance, the Institute moved the goal of economization to the background in the Appropriate Care program.

## Discussion

In the Appropriate Care program (the systematic screening of the basic benefits package), the National Health Care Institute aimed to identify and ultimately eliminate low-value care practices that were currently covered from basic health insurance as well as potential underuse, and achieve savings by improving the organization of efficiency and quality in health care delivery. The Institute’s authoritative position in the Dutch social health insurance system and its exclusive access to three national administrative datasets gave the Institute a unique position for locating low-value care on a national scale. Academic literature on disinvestment, however, shows that the right data and the authority to use them is not enough; disinvestment inevitably requires ‘clinical leadership’ [11] and ‘political will’ on the part of the stakeholders [6]. We examined how the Institute dealt with socio-political sensitivities associated with disinvestment by means of their epistemic work.

We found, first of all, that the Institute hired clinical experts in-house to create technically solid data analyses that were warranted by clinical experts and therefore were expected to be considered trustworthy. Second, to avoid stakeholders’ feelings of disempowerment and to improve technical analysis, the Institute developed a working method that engaged stakeholders in problem definition, formulating knowledge strategies and finding policy solutions. Third, the Institute created a safe space for deliberations about low-value care practices by actively avoiding the publication of information about low-value care that could be traced back to specific institutions. Fourth, the Institute shifted the goal of economization to the background and focused primarily on issues of safety and quality of care in its epistemic practices.

In summary, the Institute dealt with the socio-political sensitivities that are generally associated with such disinvestment initiatives by de-emphasizing the economization measure, de-politicizing the public deliberative space about low-value care practices, and by democratizing the epistemic practices to identify low-value care. Ultimately, this epistemic work facilitated a collaborative construction of problems relating to low-value care practices and their solutions.

### Limitations

As the scope of our fieldwork allowed us to study only the ‘Screening’ and the ‘In-depth Analysis’ Phases, this research is a limited sample of the work performed in the Appropriate Care program. Our analysis of the epistemic practices of the program shows how the Institute manages to get field parties involved in identifying low-value care and in discussing potential solutions to tackle them. However, apart from stakeholders’ involvement in the epistemic work of identifying low-value care and formulating problem solutions, successful disinvestment requires stakeholders’ engagement in implementation and monitoring. International literature reports that there is still a lack of information about effective approaches and clear procedural tools for the implementation of disinvestment policy [1, 45, 46]. Unfortunately, because the ‘Implementation’ and ‘Evaluation’ phases were still in development at the time of our fieldwork, we cannot report on the procedural tools for implementation (and their effectiveness) in the context of the Appropriate Care program. Further research is required into the epistemic practices in all four phases of the program in order to learn how these epistemic practices contribute to the actual realization of disinvestment on low-value care.

## Conclusion

Routine administrative data are no longer a source that insurers, hospitals or medical professionals draw from to account for what they themselves have done; they have become a means by which the system has started to account for itself (cf. [47]). In this paper we studied the use of administrative data by the Dutch National Health Care Institute to reduce unnecessary public expenditure by identifying and tackling low-value care currently covered from the basic benefits package. Our analysis of the Institute’s Appropriate Care program shows how the epistemic effort to identify low-value care became a co-construction between policymakers, care providers, patients and insurers of problems of ‘waste’ in the Dutch social health insurance system. Whether or not disinvestment was actually achieved, the collective epistemic work performed within the Appropriate Care program gave cognitive, moral and political standing to the idea of ‘waste’ in public health expenditure.

## Additional file

Additional file 1: Interview Topic List: See Additional file for a sample of the topic list (developed for this case study) that was used for interviews and focus groups. (DOCX 18 kb)

## Data Availability

The datasets used and/or analyzed during the current study are available from the corresponding author on reasonable request.

## Abbreviations

CRPC: Castration-Refractory Prostate Cancer
DD: Diana Delnoij
DIS: Claims Information System
EH: Eddy Houwaart
ER: Endovascular Revascularization
FM: Floortje Moes
GIP: Medicines and Medical Aids Information Project
GP: General Practitioner
HTA: Health Technology Assessment
ICD-10: International Classification of Diseases
KH: Klasien Horstman
KNGF: Royal Dutch Society for Physical Therapy
NHG: Dutch College of General Practitioners
NOV: Dutch Orthopedic Association
NvVH: The Netherlands Association of Surgeons
NVVV: The Netherlands Association for Vascular Surgery
PAD: Peripheral Artery Disease
PROM: Patient-Reported Outcome Measures
SET: Supervised Exercise Therapy
STS: Science and Technology Studies
