# Supplementary material for: Collective constructions of ‘waste’: epistemic practices for disinvestment in the context of Dutch social health insurance
Source: BMC Health Serv Res. 2019 Sep 5;19:633. doi: 10.1186/s12913-019-4434-1 (PMC6727536; doi:10.1186/s12913-019-4434-1)
Supplement: Supplementary file 1 — Interview Topic List: See Additional file for a sample of the topic list (developed for this case study) that was used for interviews and focus groups. (DOCX 18 kb) [file 12913_2019_4434_MOESM1_ESM.docx]

**Interview Topic List**

1. How did you get involved in the Appropriate Care program? What was your role/position within the context of this program?
2. What task did the Minister give you/the National Health Care Institute? (Possibly: to what extent has that task changed over the years that the project has been running?)
3. The National Health Care Institute’s task is officially to economise. How did you deal with this?
4. What types of knowledge did you need to carry out the task? How exactly did you set to work? What were the first steps in this process?
5. What role did claim data play in your work? Were the data manageable? What pitfalls did you encounter in processing the data? Can you provide us with some examples? E.g., in relation to chest pain?
6. You mentioned searching/examining the data based on a hypothesis. How does this work? How did you arrive at the hypothesis? E.g., in relation to chest pain?
7. What role do clinical guidelines and standards play in the context of the Appropriate Care program? E.g., in relation to chest pain?
8. How did you involve knowledge from daily practice/local knowledge in the process, e.g., in relation to chest pain?
9. You also used cues from those working the field about how to improve care. How did you pick up on these cues, e.g., in relation to chest pain?
10. The National Health Care Institute is very concerned about the patient’s perspective. How are patients involved in the Appropriate Care Program?
11. How did you initiate a dialogue with those working in the field? And how do you use the different knowledges (claims data/guidelines/ knowledge from daily practice/patient perspectives) in these dialogues?
12. If you were on the verge of starting the Appropriate Care program (with all the experience you gained over the past few years), would you do anything differently? Why?
13. The Appropriate Care Program is intended to generate funds and, at the same time, improve the quality of care. How does this work? How do you know whether economies are actually being made and quality is improving?
